# The genetic differences between types 1 and 2 in von Hippel-Lindau syndrome: comprehensive meta-analysis

**DOI:** 10.1186/s12886-024-03597-1

**Published:** 2024-08-13

**Authors:** Fatemeh Azimi, Masood Naseripour, Ali Aghajani, Hengameh Kasraei, Samira Chaibakhsh

**Affiliations:** 1 Eye Research Center, the Five Senses Institute, Iran University of Medical Sciences, Tehran, Iran; 2 Finetech in Medicine Research Center, Iran University of Medical Sciences, Tehran, Iran; 3 Rajaie Cardiovascular Medical and Research Center, Iran University of Medical Sciences, Tehran, Iran

**Keywords:** Von Hippel-Lindau, VHL, Genetic differences, Meta-analysis

## Abstract

**Background:**

Patients with von Hippel-Lindau (VHL) disease are at risk of developing tumors in the eye, brain, kidney, adrenal gland, and other organs based on their gene mutations. The *VHL* tumor suppressor gene contains pathogenic variants responsible for these events. This meta-analysis aims to investigate the genetic differences among the various types of VHL syndrome and their correlation with the location of mutations (exons and domains) in the *VHL* gene.

**Method:**

Papers eligible for publication until September 2023 were identified using the electronic databases of PubMed, Google Scholar, Scopus, and EMBASE. The Random Effect model was utilized to evaluate the genetic differences between type 1 and type 2 VHL syndromes.

**Results:**

The prevalence of missense mutations (MSs) was found to be 58.9% in type 1, while it was 88.1% in type 2. Interestingly, the probability of observing MSs in type 1 was 0.42 times lower compared to type 2. The mutation hotspots of the *VHL* gene were R167Q/W, Y98H, R238W, and S65L, respectively. Although type 2 had a high presentation of Y98H and R238W, it did not have a higher S65L than type 1. The analysis demonstrated a statistically significant higher prevalence of truncated mutations (PTMs) in type 1. Among type 1, large/complete deletions (L/C DELs) were found in 16.9% of cases, whereas in type 2 only 3.7%. This difference was statistically significant with a *p*-value < 0.001. Overall, the probability of identifying mutations in domain 2 compared to domain 1 was found to be 2.13 times higher in type 1 (*p*-value < 0.001). Furthermore, the probability of detecting exon 1 in comparison with observing exon 2 in type 1 was 2.11 times higher than type 2 and revealed a statistically significant result (*p*-value < 0.001). The detection of exon 2 was 2.18 times higher in type 1 (*p*-value < 0.001). In addition, the likelihood of discovering exon 2 compared with others was significantly lower in type 1 compared with type 2 VHL (OR = 0.63, *p*-value = 0.015).

**Conclusions:**

We have revealed a comprehensive genetic difference between types 1 and 2 of VHL syndrome. The significant differences in MS, PTMs, L/C DELs, and the location of the mutations between type 1 and type 2 VHL patients in the Asian, European, and American populations emphasize the genetic heterogeneity of the syndrome. These findings may pave the way for the diagnosis, treatment, and further investigation of the mechanisms behind this complex genetic disorder.

## Introduction

Von Hippel-Lindau (VHL) syndrome is a genetic disorder that is uncommon and autosomal dominant and affects approximately 1/36,000 individuals. Multiple lesions, including retinal capillary hemangioblastoma (RCH), central nervous system hemangioblastoma (CHB), renal cell carcinoma (RCC), pheochromocytomas (PCC), and as well as pancr eatic cyst (PC), kidney cysts (KC), pancreatic neuroendocrine tumors (PNETs), endolymphatic sac tumor (ELST), epididymal cyst, PGL (paragangliomas), and liver cysts have been reported in affected individuals [1, 2]. A germline pathogenic variant in the tumor suppressor gene *VHL*, located on chromosome 3p25-26 [3, 4], is found 80% of cases with a family history, while 20% of cases are de novo mutations [5]. The *VHL* gene is responsible for encoding the VHL protein (pVHL), which is a crucial determinant of the hypoxia‐inducible transcription factor α (HIF‐α) [6]. A complex consisting of elongin B, elongin C, and Cul 2 proteins is formed by pVHL. Under normal conditions, hypoxia-inducible factors (HIFs) are degraded by this complex [3]. However, when pVHL deficiency occurs, an overexpression of HIF‐α leads to unregulated angiogenesis and highly vascularized tumor development [5, 7, 8]. Nonetheless, there are still some uncertainties about these processes, and it is crucial to identify any variants of VHL syndrome to enhance the diagnosis of disease and the precision of the genetic testing.

VHL is classified into two categories: Type 1 and Type 2. Type 2 can be divided into subtypes A, B, and C. VHL disease type 1 is recognized for its low risk of PCC, but it tends to develop CHB, RCH, PC, RCC, and PNETs. On the other hand, the type 2 VHL disease is known for its high risk of PCC and is classified into type 2 A with low risk of RCC, type 2B with high risk of RCC, and type 2 C which only develops PCC (Table 1) [1, 2, 9, 10].

**Table 1:** Classification of VHL syndrome

Type	Clinical Features				Mutation
	RCH	CHB	RCC	PCC	Deletion Insertion Nonsense
**1**	✓	✓	✓	-	
**2 A**	✓	✓	-	-	**Missense**
**2B**	✓	✓	✓	-	
**2 C**	-	-	-	✓	

Many studies have demonstrated a clear connection between VHL genotypes and related phenotypes [3, 11–20]. By identifying these relationships, we could pave the way for genetic counseling and the creation of targeted therapies.

Recently, Belzutifan, a small molecule oral HIF 2 α inhibitor, has been approved by the US Food and Drug Administration for treating adults with RCC, CHB, and PNET, which is linked to VHL disease. It may be restricted to VHL patients with positive genetic tests, and the detection of *VHL* variants. Therefore, assessing or annotating each variant becomes more important for the treatment strategy of VHL disease [11].

This meta-analysis study aims to decipher the genetic differences between type 1 and type 2 VHL syndromes in a comprehensive way, which will help us understand the typical mutations in the *VHL* gene that cause VHL syndrome.

The analysis we conducted to study VHL manifestation utilized VHL disease types 1 and 2 (A, B, and C)). We examined mutation types such as missense mutations (MSs), protein-truncating mutations (PTMs) (Nonsense, frameshift, and splice site, and small insertion/deletions (INDELs) of VHL protein), large/complete deletions (L/C DELs), and variant locations (exon and domain) as described in our recent study [12].

## Methods

### Search strategy

The electronic databases PubMed, Scopus, EMBASE, and Google Scholar were searched to find papers eligible by September 2023. The keywords were selected based on MeSH terms or text words. The search syntax was: (von hippel lindau OR VHL OR von Hippel-Lindau OR *VHL* gene) AND (retinal capillary hemangioma OR RCH OR Retinal hemangioblastoma OR RH) AND (central nervous system OR CNS OR central hemangioblastoma OR CHB OR central nervous system hemangioblastoma OR CNS-HB) AND (pancreatic neuroendocrine tumor OR PNET) AND (Pancreatic cyst OR PC) AND (renal cell carcinoma OR RCC) AND (kidney cysts OR KC) AND (pheochromocytomas OR PCC) AND (Mutations OR Genotype) AND (Types OR Phenotype).

### Eligibility criteria

The inclusion criteria were: the study explored the relationship between *VHL* gene alterations and VHL syndrome; the evaluation involved at least one type of *VHL* variant or the location of the variants, and papers with available full text in English. The Exclusion criteria were: review articles, case reports, letters, and comments; and the reported information was not sufficient for analysis, as in our previous study [23].

### Data extraction

The related data was extracted and evaluated separately by two independent reviewers (FA and SCH). An Excel data sheet was created to obtain the necessary data from the documents. We entered the data for every patient because most articles reported information about people individually. The data collected includes the first author, country, continents (Asia, Europe, and America (Brazil, Mexico, and USA)), year of publication, type of variants, type of VHL disease, and the location of the mutations.

### Statistical analysis

STATA version 16 was used to conduct the statistical analysis. Odds Ratio (OR) was considered as the effect size. The analysis relevant to primary studies was utilized due to the individual cases were considered in this meta-analysis. The use of random effects model was necessary to draw inferences. The power of this model lies in its ability to analyze correlated data [21]. Our meta-analysis considers each publication as a cluster, and the cases of each publication are correlated with each other. The random effect model was employed to examine correlation. The correlation between the subjects was controlled by adding a random parameter. The model below is a typical random effect models:

*Log (π*_*ij*_*∕(1 −* π _*ij*_*)) = β0 +* β***X*** *+* ***Ui***,

The probability of having VHL phenotypes is represented by ***π***_***ij***_, ***β0*** is responsible for the intercepting, X represent the vector of independent variables, β is the parameter that controls independent variables, and random parameter ***Ui*** is responsible for the publication random effect. Adding ***Ui*** to the model makes the models consider the correlation within each publication [11]. The Odd Ratio (OR) was used to calculate the association between types of VHL, variant types, and locations. A *P* value < 0.05 was considered to be significant.

## Results

**Fig. 1:**
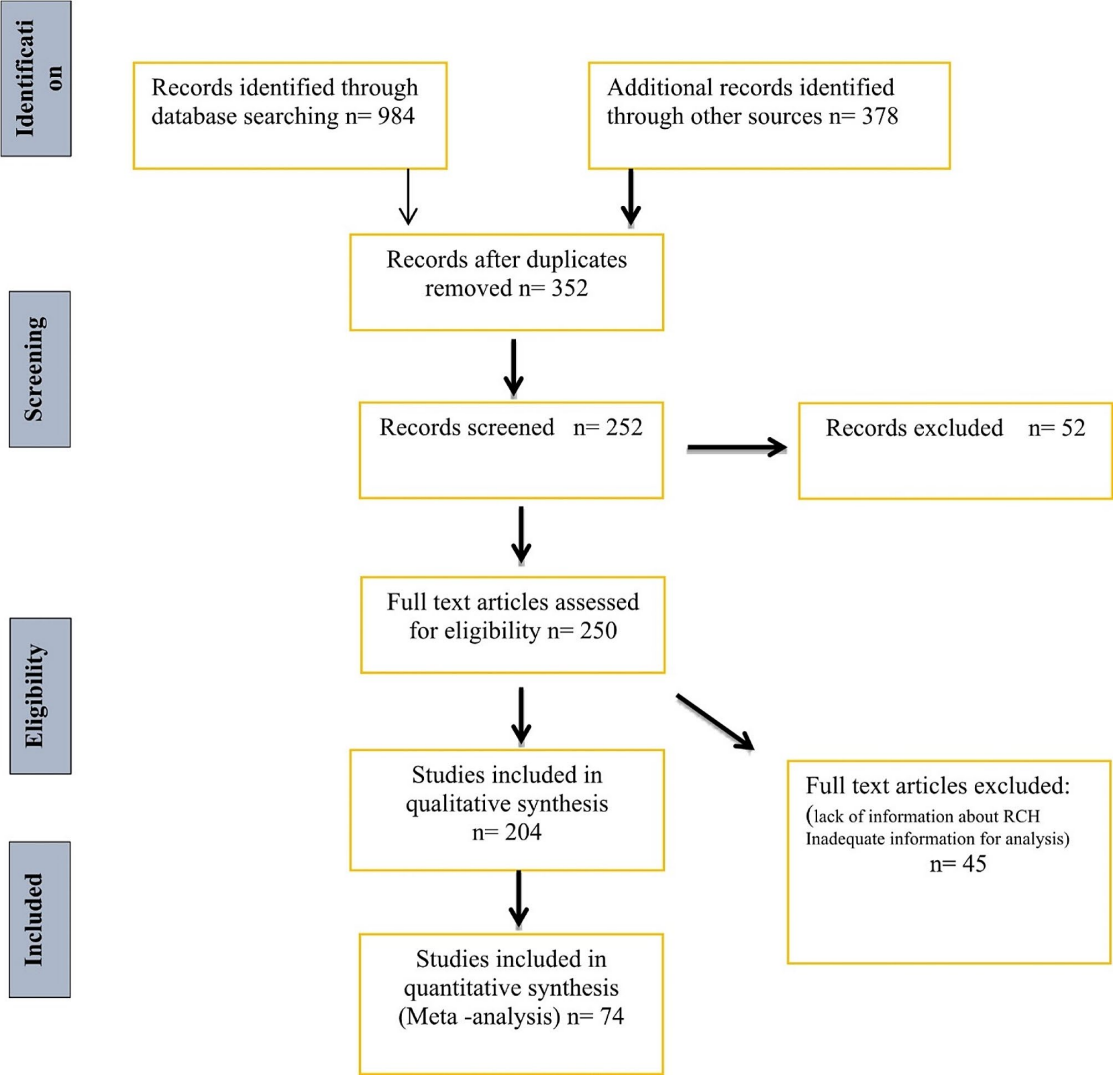
Flow diagram eligibility criteria of articles on *VHL* Mutations in VHL

**Fig. 2:**
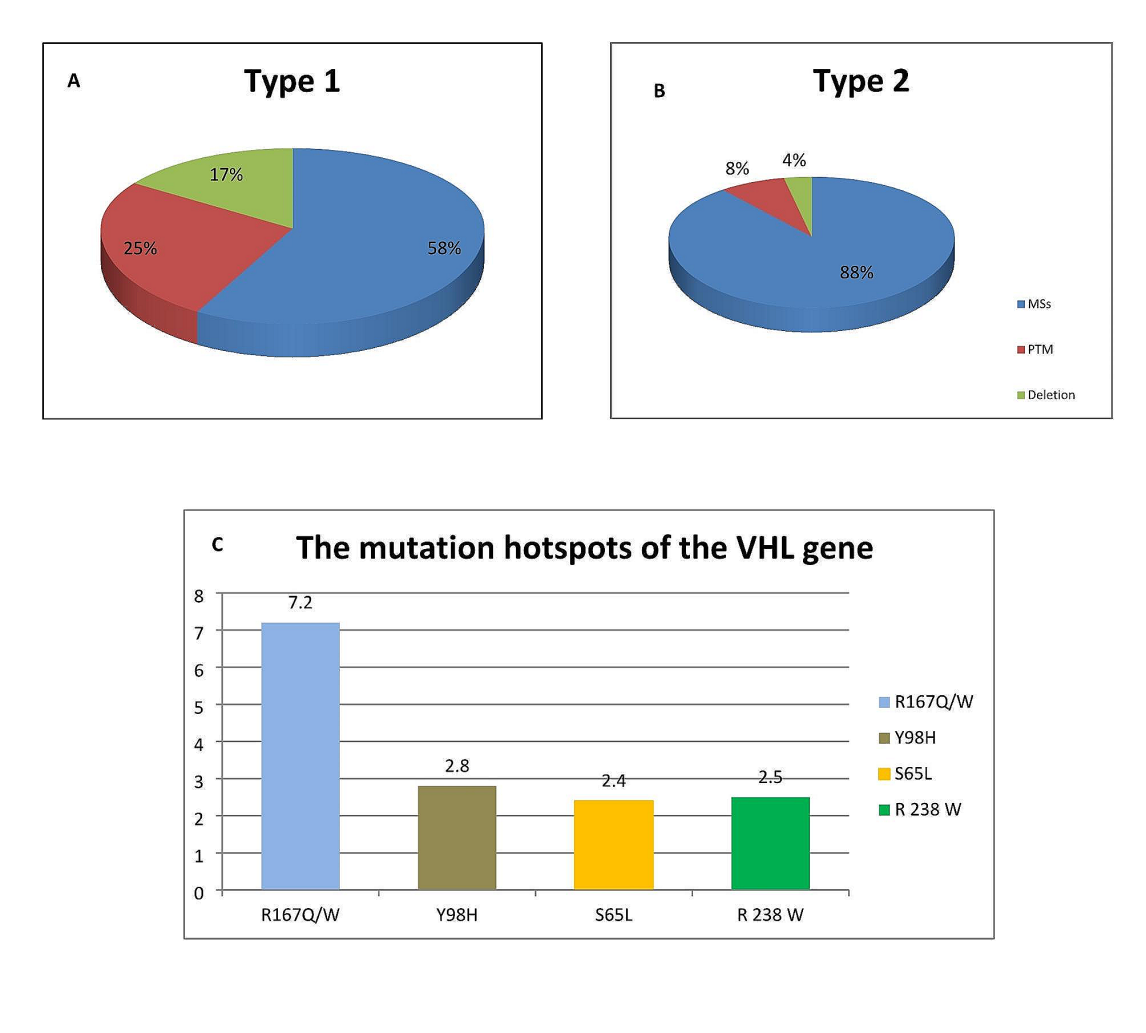
Clinical types and VHL variants. (**A**) Families whose described phenotype does not include PCC (Type1, *n* = 1630). (**B**) Families whose described phenotype includes PCC (Type 2, *n* = 631). (**C**) The frequency of the mutation hotspots of the VHL gene

The process of selecting papers is depicted in Fig. 1. This study included 74 publications that were eligible (Table 2). We analyzed data from 2261 patients according to type of VHL disease. Of these, 1630 patients (72.1%) were classified as Type 1 and 631 patients (28%) were labeled as Type 2. 25% of Type 1 cases had a PTM variant of *VHL*. On the other hand, of the 631 patients diagnosed with Type 2, MSs were found in 88% (Fig. 2, A). We found that the mutation hotspots of the *VHL* gene include S65L, R238W, Y98H, and R167Q/W, which have a frequency between 2.4 and 7.2% (Fig. 2, B). The frequency of the mutation hotspots in the *VHL* gene was shown in Fig. 2, C.

**Table 2:** Summary of data collection of *VHL* mutations in VHL disease

Region	Country	Reference	Type of Study	Sample Size	Misssense	Nonesense	Frameshift	Splice	large/complete Deletion
	**China**	Qiuli Liu (2018) [39]	Case Series	21	*				
		J. Chen (2013) [40]	Case Series	71	*			*	
		Xing Wu (2018) [41]	Case Series	4	*				
		Yulun Huang (2012) [42]	Case Series	12		*	*	*	*
		Baoan Hong (2019) [43]	Cohort	540	*	*		*	
		Pengjie Wu (2017) [44]	Case Series	36	*	*			*
		GUOBING LIN (2020) [45]	Case Series	10	*				
		Xiaosen Ma (2020) [46]	Case Series	12	*				
**Asia**	**Japan**	Kanno (1996) [47]	Case Series	25	*	*		*	
		Kenji Tamura (2023) [22]	Case Series	3	*	*			
		Keiji Iida (2004) [48]	Case Series	16	*				
		Minoru Yoshida (2000) [49]	Case Series	77	*	*	*	*	
	**Korea**	Jee-Soo Lee (2016) [50]	Case Series	13	*		*	*	*
		HIO CHUNG Kang (2005) [51]	Case Series	10	*		*		
		Hyun-Jung Cho (2009) [52]	Case Series	15	*	*	*		*
		Sang Ha Lee (2022) [53]	Retrospective	18	*	*			
		JH Kim (2013) [54]	Cohort	53	*				
	**Thailand**	Chutintorn Sriphrapradang (2017) [55]	Case Series	16	*	*			
	**India**	Nilesh Lomte (2018) [56]	Case Series	31	*				*
		Narendranath Vikkath (2015) [57]	Case Series	31	*		*	*	*
		Reshma Pandit (2016) [58]	Case Series	23	*		*		*
		Aradhana Dwivedi (2022) [59]	Case Series	31	*		*		
	**Iran**	Masood Naseripour (2023) [60]	Case Series	34	*	*	*	*	*
		Masood Naseripour (2023) [61]	Case Series	5	*				
		Shirin Hasani-Ranjbar (2009) [62]	Case Series	3	*				
	**Saudi Arabia**	Muhammad Faiyaz-Ul-Haque (2020) [63]	Case Series	5	*		*		
	**Taiwan**	J. S. Huang (2007) [64]	Case Series	6	*				
	**Israel**	Gross DJ (1996) [65]	Case Series	25	*				
**America** **Europe**	**Brazil**	Gustavo F. C. Fagundes (2019) [37]	Case Series	31	*	*	*	*	*
		Israel Gomy (1996) [66]	Case Series	20		*		*	*
		J C C Rocha (2003) [67]	Case Series	20	*		*	*	*
	**Mexico**	Oscar F Chacon-Camacho (2010) [68]	Case Series	37	*				
		ASTRID RASMUSSEN (2006) [69]	Retrospective	98	*	*	*		
	**USA**	AD Sorrell (2011) [70]	Case Series	5	*				
		Sgambati (2000) [71]	Case Series	11			*		
		DANIEL CHOO (2004) [72]	Case -control	129	*		*		*
		Catherine Stolle (1998) [73]	Case Series	73	*	*	*	*	*
		Thomas J. Manski (1997) [74]	Retrospective	347	*	*			*
		A.O Vortmeyer (1997) [75]	Case Series	24	*	*	*	*	*
		Paul A.Crossey (1995) [19]	Case Series	6	*				
		Paul A.Crossey (1994) [20]	Retrospective	116	*		*		
		John F. Bradley, (1999) [18]	Case Series	35	*				
		J. R gnarra (1994) [17]	Cohort	110	*	*	*	*	
		ATUK No (1998) [15]	Case Series	11	*				
		Sarah M. Nielsen (2011) [25]	Case Series	63	*		*		
		Anass Hajjaj (2020) [76]	Cohort	66	*		*		*
	**Netherlands**	E. Van der Harst (1998) [77]	Case Series	68	*				
		Morgan Nordstrom-O’Brien (2010) [3]	Retrospective	1548	*	*	*		*
		van Houwelingen (2005) [78]	Cohort	187					
		Frederik Hes (2000) [28]	Case Series	34					*
	**England**	Andrew R.webster (1999) [79]	Cohort	183	*		*	*	*
		Eamonn R Maher (1996) [14]	Case Series	28	*		*		
		Mary-Alice Abbott (2005) [16]	Case report	3	*				
		Emma R.Woodward (2007) [80]	Cohort	188	*		*		
		Steven Clifford (2001) [81]	Case Series	13	*		*		*
	**Poland**	Cybulski (2002) [82]	Case Series	82		*	*	*	
		Przemysław Soczomski (2021) [83]	retrospective	63	*		*	*	
	**Sweden**	Elisabeth Wittstro (2014) [84]	Case Series	13	*				*
		Joakim Crona (2014) [85]	Case-control	89	*		*		
		X Ma (2001) [86]	Case Series	47	*	*	*		
	**Central Europe**	Damjan Glavac (1996) [87]	Case Series	65	*	*	*	*	
	**Italy**	Emanuela Leonardi (2011) [88]	Cohort	426	*	*	*	*	
		Domenico Catapano (2005) [89]	Case Series	14	*				
		Penitenti (2021) [90]	Retrospective	15	*	*		*	
	**France**	Fan Chen (1995) [29]	Case Series	114	*	*	*		
		Helene Dollfus (2002) [36]	Case Series	610	*	*	*	*	*
		Alexandre Buffet (2019) [91]	Cohort	75	*				
	**Hungary**	Gergely Losonczy (2013) [92]	Case Series	7	*	*		*	
	**Bulgaria**	Maria Glushkova (2018) [93]	Case Series	5	*	*			
	**Portugal**	Marion Lenglet (2018) [94]	Case Series	13	*			*	
	**Spain**	Sergio Ruiz-Llorente (2004) [95]	Case Series	35	*	*	*		
	**Germany**	G Weirich (2009) [13]	Case Series	9	*				
		Sven Gläsker (1999) [12]	Register	99	*	*	*	*	*
	**Denmark**	Maria Bejerholm (2018) [96]	Cohort	47	*				

In type 1, the mutation hotspots Y98H and R238W were much lower than in type 2. Also, in type 2, the mutation hotspots S65L was lower than the type 1 (Table 3).

**Table 3:** The frequency of mutation hotspots of the *VHL* gene

Hotspot mutations	Type 1	Type 2 A	Type 2B	Type 2 C
**R167Q/W**	4.2	13	17.8	12.8
**Y98H**	0.1	9.4	19	2.4
**S65L**	3.3	0	0. 2	0
**R238W**	0.9	6.5	10.3	4.8

Table 4 displays the correlation between mutations and syndrome types, both globally and by continent. The analysis revealed that the prevalence of MSs was 58.9% in patients with type 1 VHL, while it was 88.1% in patients with type 2. Notably, the probability of observing MSs in type 1 patients was 0.42 times lower compared to type 2 patients, with a statistically significant *p*-value < 0.001. However, analysis demonstrated a statistically significant higher prevalence of PTMs in type 1 patients (25.3% vs. 8.4%).

**Table 4:** The correlation between mutations and types of syndrome, both in total and by continent

		Type 1 / *n* (%)	Type 2/ *n* (%)	OR	*p*-value
**Total**	**MSs**				
	yes	810(58.9)	376(88.1)	0.42	< 0.001
	no	565(41.1)	51(11.9)	1	
	**PTMs**				
	yes	348(25.3)	36(8.4)	2.01	< 0.001
	no	1027(74.7)	391(91.6)	1	
	**large/complete Deletion**				
	yes	233(16.9)	16(3.7)	2.10	< 0.001
	no	1142(83.1)	411(96.3)	1	
**Asia**	**MSs**				
	yes	307(69.1)	151(85.3)	0.70	0.036
	no	137(30.9)	26(14.7)	1	
	**PTMs**				
	yes	111(25)	20(11.3)	1.31	0.117
	no	333(75)	157(88.7)	1	
	**large/complete Deletion**				
	yes	38(8.6)	7(4)	1.66	0.179
	no	406(91.4)	170(96)	1	
**Europe**	**MSs**				
	yes	377(55.8)	148(88.6)	0.33	< 0.001
	no	299(44.2)	19(11.4)	1	
	**PTMs**				
	yes	146(21.6)	11(6.6)	2.72	< 0.001
	no	530(78.4)	156(93.4)	1	
	**large/complete Deletion**				
	yes	156(23.1)	8(4.8)	2.14	< 0.001
	no	520(76.9)	159(95.2)	1	
**America**	**MSs**				
	yes	126(49.4)	77(92.8)	0.36	0.013
	no	129(50.6)	6(7.2)	1	
	**PTMs**				
	yes	91(35.7)	5(6)	2.22	0.100
	no	164(64.3)	78(94)	1	
	**large/complete Deletion**				
	yes	39(15.3)	1(2.5)	2.77	0.002
	no	216(84.7)	82(98.8)	1	

The probability of detecting PTMs was 2.01 times higher in type 1 patients compared to type 2 patients (*p*-value < 0.001). The research revealed that there was a discrepancy in the occurrence of L/C DELs between Type 1 and type 2 patients. In type 1 patients, L/C DELs were found in 16.9% of cases, while in type 2 patients, the occurrence was only 3.7%. Additionally, there was a 2.1 times greater chance of detecting L/C DELs in type 1 patients than in type 2 patients. This difference was statistically significant with a *p*-value < 0.001.

The analysis of Asian publications and the overall data indicated a significant difference in MSs occurrence between types 1 and type 2 VHL patients. In type 1 patients, MSs were found in 69.1% of cases, while in type 2 patients it was 85.3%. Furthermore, the odds ratio (OR) for MSs in type 1 patients was found to be 0.7 when compared to type 2 patients. The statistical analysis showed a significant difference with a *p*-value of 0.036. However, no statistically significant correlation was observed between the types of VHL and the occurrence of PTMs or L/C DELs.

The prevalence of MSs was lower in type 1 patients in Europe compared to type 2 patients (55.8% vs. 88.6%). The study showed that there was a 0.33 lower chance of MSs in type 1 patients, which indicates a statistically significant difference (*p*-value < 0.001).

Analysis revealed that PTMs occurred more frequently in type 1 than in type 2 (21.6% vs. 6.6%). It was found that the likelihood of observing PTMs was 2.72 times higher, demonstrating a statistically significant OR (*p*-value < 0.001). Furthermore, type 1 patients showed a statistically significant OR of 2.14 (*p*-value < 0.001) with more frequent detection of L/C DELs (23.1 vs. 4.8%). It seemed that MSs were observed more frequently in type 2 in America compared to type 1 (92.8%vs. 49.4%), which indicated a statistically significant OR of 0.36 (*p*-value = 0.013). In addition, type 1 had a higher rate of L/C DELs than type 2 (15.3% vs. 2.5%). Analysis displayed a statistically significant OR of 2.77 (*p*-value = 0.002). Type 1 had a higher rate of PTMs than type 2 (35.7% vs. 6%), but there was no significant relation between observing PTMs and types of VHL (Table 4).

Table 5 shows the association between domains and VHL types. Detecting mutations in domain 2 was 2.13 times more likely in type 1 VHL patients than in domain 1 (*p*-value < 0.001). The probability of detecting domain 2 was 3.75 times higher in type1 VHL patients in Asians compared to domain 1, demonstrating a statistically significant relationship (*p*-value < 0.001).

**Table 5:** The relation between domains and VHL types

		Exon1	Exon2	Exon3	others
**Total**	Type1	544(39.8)	218(15.9)	502(36.7)	103(7.5)
	Type2	113(26.5)	49(11.5)	247(58)	17(4)
**Asia**	Type1	216(48.6)	57(12.8)	110(24.8)	61(13.7)
	Type2	57(32.2)	14(7.9)	102(57.6)	4(2.3)
**Europe**	Type1	224(33.4)	124(18.5)	294(43.8)	29(4.3)
	Type2	30(18.1)	14(8.4)	112(67.5)	10(6)
**America**	Type1	104(41.3)	37(14.7)	98(38.9)	13(5.2)
	Type2	26(31.3)	21(25.3)	33(39.8)	3(3.6)

Also, when investigating Europeans, the analysis revealed a statistically significant association between finding domain 1 or domain 2 and types of VHL (*p*-value = 0.012). The probability of occurrence of domain 2 was 3.23 times higher in type 1 patients compared to type 1. There was no significant relation between domains and VHL types in Americans.

Table 6 shows the result of the association between mutations in exons and types of VHL. The probability of discovering mutations in exon 1 was 2.11 times higher in type 1 than type 2 VHL patients, which led to a statistically significant result (*p*-value < 0.001). However, type 1 patients had a 0.62 times lower chance of finding mutations in exon 1 compared to type 2 (*p*-value = 0.004). A significant OR was observed when comparing the occurrence of mutations in exon 2 or exon 3 in type 1 VHL patients with type 2. Detecting mutations exon 2 was 2.18 times higher in type 1 patients (*p*-value < 0.001). Furthermore, type 1 patients were less likely to detect exon 2 mutations than type 2 patients (OR = 0.63, *p*-value = 0.015). The probability of observing mutations in exon 3 was 0.29 lower in type 1 patients than in type 2 patients (*p*-value < 0.001). In Asians, type 1 VHL patients with exon 1 mutations were 1.99 times more likely to exon 3 mutations than type 2 patients (*p*-value < 0.001).

**Table 6:** The relation between mutation in exons and types of VHL

		Domain$\:\varvec{\alpha\:}$	Domain$\:\varvec{\beta\:}$	OR	*p*-value
**Total**	Type 1	548 (37.4)	918 (62.6)	2.13	< 0.001
	Type 2	270 (48.3)	289 (51.7)		
**Asia**	Type 1	126 (29.7)	298 (70.3)	3.57	< 0.001
	Type 2	109 (57.7)	80 (42.3)		
**Europe**	Type 1	316 (40.3)	469 (59.7)	3.23	0.012
	Type 2	125 (56.6)	96 (43.4)		
**America**	Type 1	106 (41.2)	151 (58.8)	1.01	0.992
	Type 2	36 (24.2)	113 (75.8)		

Additionally, detecting mutations in exon 1 compared to others was 0.52 lower (*p*-value = 0.031). Type 1 VHL patients had a significant difference in the occurrence of mutations in either exon 2 or exon 3 compared to type 2. In type 1 patients, the probability of detecting mutations in exon 2 was 2.35 times greater (*P*-value < 0.001). The chance of observing exon 3 mutations was 0.26 times lower in type 1 patients compared to type 2 patients (*p*-value < 0.001) (Table 5). There was no other association that was significant (Table 6).

In Europe, the probability of finding mutations in exon 1 rather than exon 2 was significantly lower in type 1 VHL patients compared to type 2 by 0.78 times (*p*-value = 0.019). Also, detection of mutations in exon 1 was significantly higher in type 1 patients compared to type 2 patients (OR = 2.11, *p*-value < 0.001). Mutations in exon 1 were 0.76 less likely to occur in type 1 patients than type 2 patients (*p*-value = 0.023). Moreover, detecting mutations in exon 2 compared to exon 3 was significantly higher in type 1 patients by an OR = 2.74 (*p*-value < 0.001). Type 1 patients had significantly lower odds of finding mutations exon 3 than type 2 patients, with an OR = 0.35 (*p*-value < 0.001). No other significant association was found (Table 6).

Type 1 patients had a significant differences in exon 1 compared to exon 2, resulting in an OR of 2.04 (*p*-value = 0.041) when compared to type 2 patients. A significant OR was observed when comparing the occurrence of exon 1 mutations in type 1 VHL patients with those in type 2. Detecting mutations exon 1 was 0.39 times lower in type 1 patients (*p*-value = 0.035). The probability of finding exon 2 mutations when compared to other exons in type 1 patients was 0.19 times lower than in type 2 VHL patients, and this result was statistically significant (*p*-value = 0.023). In addition, type 1 patients had a significantly lower probability of finding mutations in exon 3 than type 2 patients (OR = 0.18, *p*-value < 0.001) (Table 6).

## Discussion

The current classification of VHL type 1 and type 2 primarily relies on clinical observations and the probability of PCC or RCC. Although studies have attempted to establish a connection between VHL genotype and phenotype, their clinical relevance is restricted. This implies that their diagnostic and therapeutic benefits are limited. However, given that patients are susceptible to tumor development throughout their lives, having a predictive outlook on the onset of VHL-related cancers could allow clinicians to devise a more personalized surveillance strategy when presented with a unique mutation.

The presented findings provide valuable insights into the genetics of VHL syndrome, specifically how different mutation types/locations related to the two distinct subtypes of VHL (type 1 and type 2).

In total, we analyzed 2,261 patients with VHL and identified 72.1% type 1 and 28% type 2, similar to the results of Tamura et al. [22]. This study revealed that mutations in the *VHL* gene are most commonly found at R167Q/W, Y98H, R238W, and S65L, respectively. Intriguingly, the mutation hotspots Y98H and R238W were present higher in type 2, especially type 2 A and 2B, which agrees with multiple studies [22–26]. Additionally, S65L was much lower in type 2 than in type 1. It is recommended that patients with mutations Y98H and R238W are likely to have several severe symptoms of the disease. Therefore, it is better to get timely screening to prevent the spread of the disease by observing this mutation in patients before they develop other symptoms.

According to this study, type 2 VHL patients have a greater chance of developing MS than type 1. On the contrary, the probability of finding L/C DELs or PTMs is almost twice as high in type 1 VHL patients, as supported by several findings [8, 27–30]. The disparities between mutation types in type 1 and type 2 VHL patients demonstrate the genetic heterogeneity of VHL syndrome and its correlation with the disease’s manifestation [25].

MSs, which often hinder the proper folding of the VHL protein, are more prevalent in type 2 VHL. This trend is true in different geographic regions. Whether the higher predisposition towards RCC or PCC is primarily attributed to the inherent nature of misfolded proteins or to the specific effects of variant MSs with higher prevalence remains uncertain. While it has been indicated that certain MSs in the *VHL* gene’s hotspot region can lead to a less stable protein that can lead to RCC development [31], but it is important to take into account the prevalence of these mutations when evaluating their overall impact. A comprehensive study of MSs variations and their prevalence could provide a more complete understanding of their overall impact on type 2 VHL. Unlike MSs, PTMs and L/C DELs that cause incomplete protein synthesis (not misshaped proteins) are observed to be less frequent in type 2 VHL.

It appears that the incomplete VHL protein has a less comparatively less detrimental effect on predisposing individuals to RCC and PCC, especially when L/C DELs occur in specific regions of the gene [32–35]. The observation that L/C DELs involving specific gene locations are less likely to result in type 2 VHL may offer an explanation for the different effects of these types of mutations in different populations.

In the Asian population, there was no significant association between L/C DELs and VHL types. Conversely, based on the available reports, L/C DELs are more prevalent in type 1 VHL among American and European populations. This suggests that Asian populations may have L/C DELs that predispose them more to RCC, however, we could not find a higher incidence of L/C DELs in the Asian population with type 2. Compared to other populations, Asians have a significantly lower prevalence of L/C DELs in Type 1. On the contrary, the higher prevalence of PTMs in type 2 VHL of Asians (compared to the other two populations) seems to be the reason why the chance of this genotype having different VHL subtypes is insignificant. Different types of PTMs in the Asian population could explain this finding, but a thorough investigation is needed to ascertain that.

The analysis of genetic mutations in Asian, European, and American populations in the context of VHL syndrome has provided a heterogeneity of VHL-associated mutations, specifically MSs, PTMs, L/C DELs, and their association with different VHL subtypes, which is similar to the study of Dollfus et al. [36] and Fagundes et al. [37].

We also demonstrated that the chance of finding a mutation in the β domain of the *VHL* gene is twice as high as finding a mutation in the α domain in type 1 VHL. Asian publications had a higher probability of domain mutation in type 1 VHL, while American publications did not have any significant odds of any domain mutation. It has been clarified that type 2 VHL is most often cause by mutations in Elongin C binding found in the α domain of the *VHL* gene, which is similar to Maxwell et al. [38]. On the other hand, mutations in the β domain that affect HIF are predominantly found in type 1 VHL. This study’s findings confirm the reported association between VHL phenotype and underlying genetic variation. However, we were unable to determine the specific mutated gene in each domain because of insufficient data. Similar findings were observed when assessing the association between exon mutations and VHL phenotypes.

Subgroup analyses by continent revealed that in the Asian population, the observance of exon 1 rather than exon 3 was significantly higher in type 1 VHL patients compared to type 2. Exon 2 mutations were significantly higher in type 1 Asian patients, while exon 3 mutations were significantly lower, suggesting that other exons play a more prominent role. The European population showed significant difference in observing exon 1 in type 1 patients compared to type 2. The likelihood of detecting exon 1 compared to exon 3 was also significantly higher in type 1 European patients. Additionally, type 1 patients were more likely to have exon 2 mutations than exon 3 mutations.

In the American population, significant associations were found between exon 1 and exon 2, with exon 1 being more prevalent among type 1 patients. In type 1 patients, there was significant decrease in the chance of detecting exon 2 compared to other exons. Furthermore, there was a significant difference in exon 3 mutations in type 1 patients compared to type 2.

In summary, mutations in exons 1, 2, and 3 have distinct associations with VHL subtypes, suggesting that the location and nature of genetic alterations within the gene may influence disease presentation.

## Conclusion

This study demonstrates that there is a significant variation in the types of mutations that are associated with different VHL subtypes in various populations. The significant differences in MSs, PTMs, L/C DELs, and the location of the mutations between type 1 and 2 VHL patients in the Asian, European, and American populations underscore the genetic heterogeneity of the syndrome. These findings emphasize the importance of considering regional and ethnic variations when studying *VHL* genetics.

The observed variations in mutation patterns between different populations may have implications for patient diagnosis and management, as well as for future research into the underlying mechanisms of VHL syndrome. Understanding these genetic differences can inform targeted therapies and personalized treatment approaches tailored to the specific mutation profiles of VHL patients in different populations.

## Data Availability

No datasets were generated or analysed during the current study.

## References

[1] Maher ER, Kaelin WG Jr. Von Hippel-Lindau disease. Medicine. 1997;76(6):381–91. 9413424 10.1097/00005792-199711000-00001

[2] Chittiboina P, Lonser RR. Von Hippel–Lindau disease. Handb Clin Neurol. 2015;132:139–56. 26564077 10.1016/B978-0-444-62702-5.00010-X PMC5121930

[3] Nordstrom-O’Brien M, van der Luijt RB, van Rooijen E, van den Ouweland AM, Majoor‐Krakauer DF, Lolkema MP, et al. Genetic analysis of Von Hippel‐Lindau disease. Hum Mutat. 2010;31(5):521–37. 20151405 10.1002/humu.21219

[4] Atik SŞ, Solmaz AE, Öztaş Z, Eğrilmez ED, Uğurlu Ş, Atik T, et al. Von Hippel-Lindau disease: the importance of retinal hemangioblastomas in diagnosis. Turkish J Ophthalmol. 2017;47(3):180. 10.4274/tjo.90912 PMC5468534 28630796

[5] Lonser RR, Glenn GM, Walther M, Chew EY, Libutti SK, Linehan WM, et al. Von Hippel-Lindau disease. Lancet. 2003;361(9374):2059–67. 12814730 10.1016/S0140-6736(03)13643-4

[6] Gossage L, Eisen T, Maher ER. VHL, the story of a tumour suppressor gene. Nat Rev Cancer. 2015;15(1):55–64. 25533676 10.1038/nrc3844

[7] Iliopoulos O, Levy AP, Jiang C, Kaelin WG Jr, Goldberg MA. Negative regulation of hypoxia-inducible genes by the Von Hippel-Lindau Protein. Proc Natl Acad Sci. 1996;93(20):10595–9. 8855223 10.1073/pnas.93.20.10595 PMC38198

[8] Tarade D, Ohh M. The HIF and other quandaries in VHL disease. Oncogene. 2018;37(2):139–47. 28925400 10.1038/onc.2017.338

[9] O’Brien F, Danapal M, Jairam S, Lalani A, Cunningham J, Morrin M, et al. Manifestations of Von Hippel Lindau syndrome: a retrospective national review. QJM: Int J Med. 2014;107(4):291–6. 10.1093/qjmed/hct249 24352051

[10] Varshney N, Kebede AA, Owusu-Dapaah H, Lather J, Kaushik M, Bhullar JS. A review of Von Hippel-Lindau syndrome. J Kidney Cancer VHL. 2017;4(3):20. 28785532 10.15586/jkcvhl.2017.88 PMC5541202

[11] Binderup MLM, Budtz-Jørgensen E, Bisgaard ML. Risk of new tumors in Von Hippel–Lindau patients depends on age and genotype. Genet Sci. 2016;18(1):89–97. 10.1038/gim.2015.44 25834951

[12] Gläsker S, Bender BU, Apel TW, Natt E, van Velthoven V, Scheremet R, et al. The impact of molecular genetic analysis of theVHL gene in patients with haemangioblastomas of the central nervous system. J Neurol Neurosurg Psychiatry. 1999;67(6):758–62. 10567493 10.1136/jnnp.67.6.758 PMC1736691

[13] Weirich G, Klein B, Wöhl T, Engelhardt D, Brauch H. VHL2C phenotype in a German von Hippel-Lindau family with concurrent VHL germline mutations P81S and L188V. J Clin Endocrinol Metabolism. 2002;87(11):5241–6. 10.1210/jc.2002-020651 12414898

[14] Maher ER, Webster AR, Richards FM, Green JS, Crossey PA, Payne SJ, et al. Phenotypic expression in Von Hippel-Lindau disease: correlations with germline VHL gene mutations. J Med Genet. 1996;33(4):328–32. 8730290 10.1136/jmg.33.4.328 PMC1050584

[15] Atuk NO, Stolle C, Owen JA Jr, Carpenter JT, Vance ML. Pheochromocytoma in Von Hippel-Lindau disease: clinical presentation and mutation analysis in a large, multigenerational kindred. J Clin Endocrinol Metabolism. 1998;83(1):117–20. 10.1210/jcem.83.1.4479 9435426

[16] Abbott MA, Nathanson KL, Nightingale S, Maher ER, Greenstein RM. The Von Hippel–Lindau (VHL) germline mutation V84L manifests as early-onset bilateral pheochromocytoma. Am J Med Genet Part A. 2006;140(7):685–90. 16502427 10.1002/ajmg.a.31116

[17] Gnarra J, Tory K, Weng Y, Schmidt L, Wei M, Li H, et al. Mutations of the VHL tumour suppressor gene in renal carcinoma. Nat Genet. 1994;7(1):85–90. 7915601 10.1038/ng0594-85

[18] Bradley JF, Collins DL, Schimke RN, Parrott HN, Rothberg PG. Two distinct phenotypes caused by two different missense mutations in the same codon of the VHL gene. Am J Med Genet. 1999;87(2):163–7. 10533030 10.1002/(SICI)1096-8628(19991119)87:2<163::AID-AJMG7>3.0.CO;2-A

[19] Crossey P, Eng C, Ginalska-Malinowska M, Lennard T, Wheeler D, Ponder B, et al. Molecular genetic diagnosis of Von Hippel-Lindau disease in familial phaeochromocytoma. J Med Genet. 1995;32(11):885–6. 8592333 10.1136/jmg.32.11.885 PMC1051741

[20] Crossey PA, Foster K, Richards FM, Phipps ME, Latif F, Tory K, et al. Molecular genetic investigations of the mechanism of tumourigenesis in Von Hippel-Lindau disease: analysis of allele loss in VHL tumours. Hum Genet. 1994;93:53–8. 8270255 10.1007/BF00218913

[21] Fitzmaurice GM, Laird NM, Ware JH. Applied longitudinal analysis: Wiley; 2012.

[22] Tamura K, Kanazashi Y, Kawada C, Sekine Y, Maejima K, Ashida S, et al. Variant spectrum of Von Hippel–Lindau disease and its genomic heterogeneity in Japan. Hum Mol Genet. 2023;32(12):2046–54. 36905328 10.1093/hmg/ddad039 PMC10244221

[23] Azimi F, Aghajani A, Khakpour G, Chaibakhsh S. A meta-analysis of different von Hippel Lindau mutations: are they related to retinal capillary hemangioblastoma? Mol Genet Genomics. 2022;297(6):1615–26. 36006455 10.1007/s00438-022-01940-z

[24] Liu P, Zhu F, Li M, Dube DA, Liu Q, Wang C, et al. Von Hippel-Lindau Black Forest mutation inherited in a large Chinese family. Gland Surg. 2019;8(4):343. 31538058 10.21037/gs.2019.08.03 PMC6723002

[25] Nielsen SM, Rubinstein WS, Thull DL, Armstrong MJ, Feingold E, Stang MT, et al. Genotype–phenotype correlations of pheochromocytoma in two large Von Hippel–Lindau (VHL) type 2A kindreds with different missense mutations. Am J Med Genet Part A. 2011;155(1):168–73. 10.1002/ajmg.a.33760 PMC3085839 21204227

[26] Brauch H, Kishida T, Glavac D, Chen F, Pausch F, Höfler H, et al. Von Hippel-Lindau (VHL) disease with pheochromocytoma in the Black Forest region of Germany: evidence for a founder effect. Hum Genet. 1995;95:551–6. 7759077 10.1007/BF00223868

[27] Ong KR, Woodward ER, Killick P, Lim C, Macdonald F, Maher ER. Genotype–phenotype correlations in Von Hippel-Lindau disease. Hum Mutat. 2007;28(2):143–9. 17024664 10.1002/humu.20385

[28] Hes F, Zewald R, Peeters T, Sijmons R, Links T, Verheij J, et al. Genotype-phenotype correlations in families with deletions in the Von Hippel-Lindau (VHL) gene. Hum Genet. 2000;106:425–31. 10830910 10.1007/s004390000265

[29] Chen F, Kishida T, Yao M, Hustad T, Glavac D, Dean M, et al. Germline mutations in the Von Hippel–Lindau disease tumor suppressor gene: correlations with phenotype. Hum Mutat. 1995;5(1):66–75. 7728151 10.1002/humu.1380050109

[30] Nielsen SM, Rhodes L, Blanco IG, Chung WK, Eng C, Maher ER, et al. editors. Von Hippel-Lindau disease: genetics and role of genetic counseling in a multiple neoplasia syndrome2016: American Society of Clinical Oncology. 10.1200/JCO.2015.65.6140 27114602

[31] Fields FR, Suresh N, Hiller M, Freed SD, Haldar K, Lee SW. Algorithmic assessment of missense mutation severity in the Von-Hippel Lindau protein. PLoS ONE. 2020;15(11):e0234100. 33151962 10.1371/journal.pone.0234100 PMC7644048

[32] Maranchie JK, Afonso A, Albert PS, Kalyandrug S, Phillips JL, Zhou S, et al. Solid renal tumor severity in Von Hippel Lindau disease is related to germline deletion length and location. Hum Mutat. 2004;23(1):40–6. 14695531 10.1002/humu.10302

[33] Cascón A, Escobar B, Montero-Conde C, Rodríguez‐Antona C, Ruiz‐Llorente S, Osorio A, et al. Loss of the actin regulator HSPC300 results in clear cell renal cell carcinoma protection in Von Hippel‐Lindau patients. Hum Mutat. 2007;28(6):613–21. 17311301 10.1002/humu.20496

[34] Franke G, Bausch B, Hoffmann MM, Cybulla M, Wilhelm C, Kohlhase J, et al. Alu-Alu recombination underlies the vast majority of large VHL germline deletions: molecular characterization and genotype–phenotype correlations in VHL patients. Hum Mutat. 2009;30(5):776–86. 19280651 10.1002/humu.20948

[35] McNeill A, Rattenberry E, Barber R, Killick P, MacDonald F, Maher ER. Genotype–phenotype correlations in VHL exon deletions. Am J Med Genet Part A. 2009;149(10):2147–51. 10.1002/ajmg.a.33023 19764026

[36] Dollfus Hln, Massin P, Taupin P, Nemeth C, Amara S, Giraud S, et al. Retinal hemangioblastoma in Von Hippel-Lindau disease: a clinical and molecular study. Investig Ophthalmol Vis Sci. 2002;43(9):3067–74. 12202531

[37] Fagundes GF, Petenuci J, Lourenco DM Jr, Trarbach EB, Pereira MAA, Correa D’Eur JE, et al. New insights into pheochromocytoma surveillance of young patients with VHL missense mutations. J Endocr Soc. 2019;3(9):1682–92. 31528828 10.1210/js.2019-00225 PMC6735756

[38] Maxwell PH, Wiesener MS, Chang G-W, Clifford SC, Vaux EC, Cockman ME, et al. The tumour suppressor protein VHL targets hypoxia-inducible factors for oxygen-dependent proteolysis. Nature. 1999;399(6733):271–5. 10353251 10.1038/20459

[39] Liu Q, Yuan G, Tong D, Liu G, Yi Y, Zhang J, et al. Novel genotype–phenotype correlations in five Chinese families with Von Hippel–Lindau disease. Endocr Connections. 2018;7(7):870–8. 10.1530/EC-18-0167 PMC6026882 29871882

[40] Chen J, Geng W, Zhao Y, Zhao H, Wang G, Huang F, et al. Clinical and mutation analysis of four Chinese families with Von Hippel-Lindau disease. Clin Transl Oncol. 2013;15(5):391–7. 23143947 10.1007/s12094-012-0940-x

[41] Wu X, Chen L, Zhang Y, Xie H, Xue M, Wang Y, et al. A novel mutation in the VHL gene in a Chinese family with Von Hippel-Lindau Disease. BMC Med Genet. 2018;19(1):1–5. 30477447 10.1186/s12881-018-0716-4 PMC6258150

[42] Huang Y, Zhou D, Liu J, Zhou P, Li X, Wang Z. Germline mutations of the VHL gene in seven Chinese families with Von Hippel-Lindau disease. Int J Mol Med. 2012;29(1):47–52. 21972040 10.3892/ijmm.2011.808

[43] Hong B, Ma K, Zhou J, Zhang J, Wang J, Liu S, et al. Frequent mutations of VHL Gene and the clinical phenotypes in the Largest Chinese Cohort with Von Hippel–Lindau Disease. Front Genet. 2019;10:867. 31620170 10.3389/fgene.2019.00867 PMC6759728

[44] Peng S, Shepard MJ, Wang J, Li T, Ning X, Cai L, et al. Genotype-phenotype correlations in Chinese Von Hippel–Lindau disease patients. Oncotarget. 2017;8(24):38456. 28388566 10.18632/oncotarget.16594 PMC5503545

[45] Lin G, Zhao Y, Zhang Z, Zhang H. Clinical diagnosis, treatment and screening of the VHL gene in three Von Hippel–Lindau disease pedigrees. Experimental Therapeutic Med. 2020;20(2):1237–44. 10.3892/etm.2020.8829 PMC7388314 32742360

[46] Ma X, Li M, Tong A, Wang F, Cui Y, Zhang X, et al. Genetic and clinical profiles of pheochromocytoma and paraganglioma: a single center study. Front Endocrinol. 2020;11:574662. 10.3389/fendo.2020.574662 PMC7761866 33362715

[47] Kanno H, Shuin T, Kondo K, Ito S, Hosaka M, Torigoe S, et al. Molecular genetic diagnosis of Von Hippel-Lindau disease: analysis of five Japanese families. Jpn J Cancer Res. 1996;87(5):423–8. 8641976 10.1111/j.1349-7006.1996.tb00240.x PMC5921130

[48] Iida K, Okimura Y, Takahashi K, Inomata S, Iguchi G, Kaji H, et al. A variety of phenotype with R161Q germline mutation of the Von Hippel-Lindau tumor suppressor gene in Japanese kindred. Int J Mol Med. 2004;13(3):401–4. 14767570

[49] Yoshida M, Ashida S, Kondo K, Kobayashi K, Kanno H, Shinohara N, et al. Germ-line mutation analysis in patients with Von Hippel‐Lindau disease in Japan: an extended study of 77 families. Jpn J Cancer Res. 2000;91(2):204–12. 10761708 10.1111/j.1349-7006.2000.tb00933.x PMC5926327

[50] Lee J-S, Lee J-H, Lee KE, Kim JH, Hong JM, Ra EK, et al. Genotype-phenotype analysis of Von Hippel-Lindau syndrome in Korean families: HIF-α binding site missense mutations elevate age-specific risk for CNS hemangioblastoma. BMC Med Genet. 2016;17(1):1–8. 27439424 10.1186/s12881-016-0306-2 PMC4955248

[51] Kang HC, Kim I-J, Park J-H, Shin Y, Jang S-G, Ahn S-A, et al. Three novel VHL germline mutations in Korean patients with Von Hippel-Lindau disease and pheochromocytomas. Oncol Rep. 2005;14(4):879–83. 16142346

[52] Cho H-J, Ki C-S, Kim J-W. Improved detection of germline mutations in Korean VHL patients by multiple ligation-dependent probe amplification analysis. J Korean Med Sci. 2009;24(1):77–83. 19270817 10.3346/jkms.2009.24.1.77 PMC2650969

[53] Lee SH, Park KH, Woo SJ, Park SJ, Joo K. Clinical and genetic characteristics of retinal capillary hemangioblastoma in Korean patients. Korean J Ophthalmology: KJO. 2022;36(6):543. 36281577 10.3341/kjo.2022.0079 PMC9745345

[54] Kim J, Seong MW, Lee K, Choi H, Ku E, Bae J, et al. Germline mutations and genotype–phenotype correlations in patients with apparently sporadic pheochromocytoma/paraganglioma in Korea. Clin Genet. 2014;86(5):482–6. 24134185 10.1111/cge.12304

[55] Sriphrapradang C, Choopun K, Tunteeratum A, Sura T. Genotype-phenotype correlation in patients with germline mutations of VHL, RET, SDHB, and SDHD genes: Thai experience. Clin Med Insights: Endocrinol Diabetes. 2017;10:1179551417705122. 28469506 10.1177/1179551417705122 PMC5404897

[56] Lomte N, Kumar S, Sarathi V, Pandit R, Goroshi M, Jadhav S, et al. Genotype phenotype correlation in Asian Indian Von Hippel–Lindau (VHL) syndrome patients with pheochromocytoma/paraganglioma. Fam Cancer. 2018;17(3):441–9. 29124493 10.1007/s10689-017-0058-y

[57] Vikkath N, Valiyaveedan S, Nampoothiri S, Radhakrishnan N, Pillai GS, Nair V, et al. Genotype–phenotype analysis of Von Hippel–Lindau syndrome in fifteen Indian families. Fam Cancer. 2015;14(4):585–94. 25952756 10.1007/s10689-015-9806-z

[58] Pandit R, Khadilkar K, Sarathi V, Kasaliwal R, Goroshi M, Khare S, et al. Germline mutations and genotype–phenotype correlation in Asian Indian patients with pheochromocytoma and paraganglioma. Eur J Endocrinol. 2016;175(4):311–23. 27539324 10.1530/EJE-16-0126

[59] Dwivedi A, Moirangthem A, Pandey H, Sharma P, Srivastava P, Yadav P, et al. Von Hippel–Lindau (VHL) disease and VHL-associated tumors in Indian subjects: VHL gene testing in a resource constraint setting. Egypt J Med Hum Genet. 2022;23(1):126. 10.1186/s43042-022-00338-1

[60] Naseripour M, Azimi F, Talebi S, Mirshahi R, Kiaee R, Sedaghat A, et al. Investigation of germline VHL variants in Iranian patients with retinal capillary hemangioblastoma and genotype-phenotype analysis. Ophthalmic Genet. 2023;44(3):211–7. 36715412 10.1080/13816810.2022.2138455

[61] Naseripour M, Bagherzadeh K, Khakpoor GG, Sedaghat A, Mirshahi R, Kasraei H et al. Novel VHL Germline Mutations in Iranian RCH Patients. 2023.

[62] Hasani-Ranjbar S, Amoli MM, Ebrahim-Habibi A, Haghpanah V, Hejazi M, Soltani A, et al. Mutation screening of VHL gene in a family with malignant bilateral pheochromocytoma: from isolated familial pheochromocytoma to Von Hippel-Lindau disease. Fam Cancer. 2009;8:465–71. 19649731 10.1007/s10689-009-9266-4

[63] Faiyaz-Ul-Haque M, Jamil M, Aslam M, Abalkhail H, Al-Dayel F, Basit S, et al. Novel and recurrent germline mutations in the VHL gene in 5 arab patients with Von Hippel-Lindau disease. Cancer Genet. 2020;243:1–6. 32179488 10.1016/j.cancergen.2020.02.006

[64] Huang J, Huang C, Chen S, Chien C, Chen C, Lin C. Associations between VHL genotype and clinical phenotype in familial Von Hippel–Lindau disease. Eur J Clin Invest. 2007;37(6):492–500. 17537157 10.1111/j.1365-2362.2007.01806.x

[65] Gross DJ, Avishai N, Meiner V, Filon D, Zbar B, Abeliovich D. Familial pheochromocytoma associated with a novel mutation in the Von Hippel-Lindau gene. J Clin Endocrinol Metabolism. 1996;81(1):147–9. 10.1210/jcem.81.1.8550742 8550742

[66] Gomy I, Molfetta GA, de Andrade Barreto E, Ferreira CA, Zanette DL, Casali-da-Rocha JC, et al. Clinical and molecular characterization of Brazilian families with Von Hippel-Lindau disease: a need for delineating genotype-phenotype correlation. Fam Cancer. 2010;9(4):635–42. 20567917 10.1007/s10689-010-9357-2

[67] Rocha J, Silva R, Mendonca B, Marui S, Simpson A, Camargo A. High frequency of novel germline mutations in the VHL gene in the heterogeneous population of Brazil. J Med Genet. 2003;40(3):e31–e. 12624160 10.1136/jmg.40.3.e31 PMC1735383

[68] Chacon-Camacho OF, Rodriguez‐Dennen F, Camacho‐Molina A, Rasmussen A, Alonso‐Vilatela E, Zenteno JC. Clinical and molecular features of familial and sporadic cases of Von Hippel‐Lindau disease from Mexico. Clin Exp Ophthalmol. 2010;38(3):277–83. 20447124 10.1111/j.1442-9071.2010.02241.x

[69] Rasmussen A, Nava-Salazar S, Yescas P, Alonso E, Revuelta R, Ortiz I, et al. Von Hippel–Lindau disease germline mutations in Mexican patients with cerebellar hemangioblastoma. J Neurosurg. 2006;104(3):389–94. 16572651 10.3171/jns.2006.104.3.389

[70] Sorrell AD, Lee S, Stolle C, Ellenhorn J, Grix A, Kaelin WG Jr, et al. Clinical and functional properties of novel VHL mutation (X214L) consistent with type 2A phenotype and low risk of renal cell carcinoma. Clin Genet. 2011;79(6):539–45. 20560986 10.1111/j.1399-0004.2010.01464.x PMC2958253

[71] Sgambati M, Stolle C, Choyke P, Walther M, Zbar B, Linehan W, et al. Mosaicism in Von Hippel–Lindau disease: lessons from kindreds with germline mutations identified in offspring with mosaic parents. Am J Hum Genet. 2000;66(1):84–91. 10631138 10.1086/302726 PMC1288351

[72] Choo D, Shotland L, Mastroianni M, Glenn G, van Waes C, Linehan WM, et al. Endolymphatic sac tumors in Von Hippel—Lindau disease. J Neurosurg. 2004;100(3):480–7. 15035284 10.3171/jns.2004.100.3.0480

[73] Stolle C, Glenn G, Zbar B, Humphrey JS, Choyke P, Walther M, et al. Improved detection of germline mutations in the Von Hippel-Lindau disease tumor suppressor gene. Hum Mutat. 1998;12(6):417–23. 9829911 10.1002/(SICI)1098-1004(1998)12:6<417::AID-HUMU8>3.0.CO;2-K

[74] Manski TJ, Heffner DK, Glenn GM, Patronas NJ, Pikus AT, Katz D, et al. Endolymphatic sac tumors: a source of morbid hearing loss in Von Hippel-Lindau disease. JAMA. 1997;277(18):1461–6. 9145719 10.1001/jama.1997.03540420057030

[75] Vortmeyer AO, Lubensky IA, Fogt F, Linehan WM, Khettry U, Zhuang Z. Allelic deletion and mutation of the Von Hippel-Lindau (VHL) tumor suppressor gene in pancreatic microcystic adenomas. Am J Pathol. 1997;151(4):951. 9327728 PMC1858030

[76] Hajjaj A, van Overdam KA, Oldenburg RA, Koopmans AE, van den Ouweland AM, de Klein A, et al. Retinal haemangioblastomas in Von Hippel–Lindau germline mutation carriers: progression, complications and treatment outcome. Acta Ophthalmol. 2020;98(5):464–71. 32003155 10.1111/aos.14360 PMC7496349

[77] Van der Harst E, De Krijger R, Dinjens W, Weeks L, Bonjer H, Bruining H, et al. Germline mutations in the vhl gene in patients presenting with phaeochromocytomas. Int J Cancer. 1998;77(3):337–40. 9663592 10.1002/(SICI)1097-0215(19980729)77:3<337::AID-IJC5>3.0.CO;2-P

[78] van Houwelingen KP, van Dijk BA, Hulsbergen-van de Kaa CA, Schouten LJ, Gorissen HJ, Schalken JA, et al. Prevalence of Von Hippel-Lindau gene mutations in sporadic renal cell carcinoma: results from the Netherlands cohort study. BMC Cancer. 2005;5:1–11. 15932632 10.1186/1471-2407-5-57 PMC1177929

[79] Webster AR, Maher ER, Moore AT. Clinical characteristics of ocular angiomatosis in Von Hippel-Lindau disease and correlation with germline mutation. Arch Ophthalmol. 1999;117(3):371–8. 10088816 10.1001/archopht.117.3.371

[80] Woodward ER, Buchberger A, Clifford SC, Hurst LD, Affara NA, Maher ER. Comparative sequence analysis of the VHL tumor suppressor gene. Genomics. 2000;65(3):253–65. 10857749 10.1006/geno.2000.6144

[81] Clifford SC, Cockman ME, Smallwood AC, Mole DR, Woodward ER, Maxwell PH, et al. Contrasting effects on HIF-1α regulation by disease-causing pVHL mutations correlate with patterns of tumourigenesis in Von Hippel-Lindau disease. Hum Mol Genet. 2001;10(10):1029–38. 11331613 10.1093/hmg/10.10.1029

[82] Cybulski C, Krzystolik K, Murgia A, Gorski B, Dębniak T, Jakubowska A, et al. Germline mutations in the Von Hippel-Lindau (VHL) gene in patients from Poland: disease presentation in patients with deletions of the entire VHL gene. J Med Genet. 2002;39(7):e38–e. 12114495 10.1136/jmg.39.7.e38 PMC1735187

[83] Soczomski P, Jurecka-Lubieniecka B, Krzywon A, Cortez AJ, Zgliczynski S, Rogozik N, et al. A direct comparison of patients with hereditary and sporadic pancreatic neuroendocrine tumors: evaluation of clinical course, prognostic factors and genotype–phenotype correlations. Front Endocrinol. 2021;12:681013. 10.3389/fendo.2021.681013 PMC8194819 34122352

[84] Wittström E, Nordling M, Andréasson S. Genotype-phenotype correlations, and retinal function and structure in Von Hippel-Lindau disease. Ophthalmic Genet. 2014;35(2):91–106. 24555745 10.3109/13816810.2014.886265

[85] Crona J, Nordling M, Maharjan R, Granberg D, Stålberg P, Hellman P, et al. Integrative genetic characterization and phenotype correlations in pheochromocytoma and paraganglioma tumours. PLoS ONE. 2014;9(1):e86756. 24466223 10.1371/journal.pone.0086756 PMC3899286

[86] Ma X, Yang K, Lindblad P, Egevad L, Hemminki K. VHL gene alterations in renal cell carcinoma patients: novel hotspot or founder mutations and linkage disequilibrium. Oncogene. 2001;20(38):5393–400. 11536052 10.1038/sj.onc.1204692

[87] Glavač D, Neumann HP, Wittke C, Jaenig H, Mašek O, Streicher T, et al. Mutations in the VHL tumor suppressor gene and associated lesions in families with Von Hippel-Lindau disease from central Europe. Hum Genet. 1996;98(3):271–80. 8707293 10.1007/s004390050206

[88] Leonardi E, Martella M, Tosatto SC, Murgia A. Identification and in silico analysis of novel Von Hippel-Lindau (VHL) gene variants from a large population. Ann Hum Genet. 2011;75(4):483–96. 21463266 10.1111/j.1469-1809.2011.00647.x

[89] Catapano D, Muscarella LA, Guarnieri V, Zelante L, D’Angelo VA, D’Agruma L. Hemangioblastomas of central nervous system: molecular genetic analysis and clinical management. Neurosurgery. 2005;56(6):1215–21. 15918937 10.1227/01.NEU.0000159646.15026.D6

[90] Penitenti F, Landoni L, Scardoni M, Piredda M, Cingarlini S, Scarpa A, et al. Clinical presentation, genotype–phenotype correlations, and outcome of pancreatic neuroendocrine tumors in Von Hippel–Lindau syndrome. Endocrine. 2021;74(1):180–7. 34036514 10.1007/s12020-021-02752-8 PMC8440302

[91] Buffet A, Ben Aim L, Leboulleux S, Drui D, Vezzosi D, Libé R, et al. Positive impact of genetic test on the management and outcome of patients with paraganglioma and/or pheochromocytoma. J Clin Endocrinol Metabolism. 2019;104(4):1109–18. 10.1210/jc.2018-02411 30698717

[92] Losonczy G, Fazakas F, Pfliegler G, Komáromi I, Balázs E, Pénzes K, et al. Three novel germ-line VHL mutations in Hungarian Von Hippel-Lindau patients, including a nonsense mutation in a fifteen-year-old boy with renal cell carcinoma. BMC Med Genet. 2013;14(1):1–8. 23298237 10.1186/1471-2350-14-3 PMC3556325

[93] Glushkova M, Dimova P, Yordanova I, Todorov T, Tourtourikov I, Mitev V, et al. Molecular-genetic diagnostics of Von Hippel-Lindau syndrome (VHL) in Bulgaria: first complex mutation event in the VHL gene. Int J Neurosci. 2018;128(2):117–24. 28849724 10.1080/00207454.2017.1372436

[94] Lenglet M, Robriquet F, Schwarz K, Camps C, Couturier A, Hoogewijs D, et al. Identification of a new VHL exon and complex splicing alterations in familial erythrocytosis or Von Hippel-Lindau disease. Blood J Am Soc Hematol. 2018;132(5):469–83. 10.1182/blood-2018-03-838235 29891534

[95] Ruiz-Llorente S, Bravo J, Cebrián A, Cascón A, Pollan M, Tellería D, et al. Genetic characterization and structural analysis of VHL Spanish families to define genotype–phenotype correlations. Hum Mutat. 2004;23(2):160–9. 14722919 10.1002/humu.10309

[96] Christensen MB, Wadt K, Jensen UB, Lautrup CK, Bojesen A, Krogh LN, et al. Exploring the hereditary background of renal cancer in Denmark. PLoS ONE. 2019;14(4):e0215725. 31034483 10.1371/journal.pone.0215725 PMC6488054

